# Supplementary material for: Adherence to Clinical Practice Guidelines and Colorectal Cancer Survival: A Retrospective High-Resolution Population-Based Study in Spain
Source: Int J Environ Res Public Health. 2020 Sep 14;17(18):6697. doi: 10.3390/ijerph17186697 (PMC7558406; doi:10.3390/ijerph17186697)
Supplement: Supplementary file 1 [file ijerph-17-06697-s001.pdf]

Table S1. Distribution of colorectal cancer patients according to the combination of treatments received.

	Total		TNM7 Stage									
			I		II		III		IV		Unknown	
	n	(%)	n	(%)	n	(%)	n	(%)	n	(%)	n	(%)
**Total**	**1050**	**(100.0)**	**179**	**(100.0)**	**278**	**(100.0)**	**277**	**(100.0)**	**264**	**(100.0)**	**52**	**(100.0)**
Untreated	95	(9.0)	5	(2.8)	10	(3.6)	2	(.7)	57	(21.6)	21	(40.4)
Only surgery	423	(40.3)	143	(79.9)	180	(64.7)	57	(20.6)	28	(10.6)	15	(28.8)
Only chemotherapy	35	(3.3)	0	(.0)	0	(.0)	1	(.4)	34	(12.9)	0	(.0)
Only radiotherapy	11	(1.0)	2	(1.1)	0	(.0)	4	(1.4)	3	(1.1)	2	(3.8)
Surgery+Chemo	237	(22.6)	3	(1.7)	45	(16.2)	124	(44.8)	64	(24.2)	1	(1.9)
Surgery+Radio	17	(1.6)	0	(.0)	9	(3.2)	7	(2.5)	1	(.4)	0	(.0)
Chemo+Radio	11	(1.0)	2	(1.1)	3	(1.1)	4	(1.4)	2	(.8)	0	(.0)
Surgery+Chemo+Radio	136	(13.0)	22	(12.3)	29	(10.4)	71	(25.6)	12	(4.5)	2	(3.8)
Target treatment*	62	(5.9)	1	(.6)	0	(.0)	1	(.4)	60	(22.7)	0	(.0)
Unknown	23	(2.2)	1	(.6)	2	(.7)	6	(2.2)	3	(1.1)	11	(21.2)

(*) 100% combined with chemotherapy; 71% combined with surgery; 13% combined with radiotherapy

Table S2. Distribution of colon cancer patients according to the combination of treatments received.

	Total		TNM7 Stage									
			I		II		III		IV		Unknown	
	n	(%)	n	(%)	n	(%)	n	(%)	n	(%)	n	(%)
**Total**	**697**	**(100.0)**	**111**	**(100.0)**	**203**	**(100.0)**	**166**	**(100.0)**	**183**	**(100.0)**	**34**	**(100.0)**
Untreated	67	(9.6)	3	(2.7)	9	(4.4)	1	(.6)	39	(21.3)	15	(44.1)
Only surgery	337	(48.4)	105	(94.6)	150	(73.9)	49	(29.5)	22	(12.0)	11	(32.4)
Only chemotherapy	21	(3.0)	0	(.0)	0	(.0)	1	(.6)	20	(10.9)	0	(.0)
Only radiotherapy	0	(.0)	0	(.0)	0	(.0)	0	(.0)	0	(.0)	0	(.0)
Surgery+Chemo	211	(30.3)	2	(1.8)	42	(20.7)	111	(66.9)	55	(30.1)	1	(2.9)
Surgery+Radio	1	(.1)	0	(.0)	1	(.5)	0	(.0)	0	(.0)	0	(.0)
Chemo+Radio	1	(.1)	0	(.0)	0	(.0)	0	(.0)	1	(.5)	0	(.0)
Surgery+Chemo+Radio	1	(.1)	0	(.0)	0	(.0)	0	(.0)	1	(.5)	0	(.0)
Target treatment*	45	(6.5)	1	(.9)	0	(.0)	1	(.6)	43	(23.5)	0	(.0)
Unknown	13	(1.9)	0	(.0)	1	(.5)	3	(1.8)	2	(1.1)	7	(20.6)

(*) 100% combined with chemotherapy; 67% combined with surgery; 2% combined with radiotherapy

Table S3. Distribution of rectal cancer patients according to the combination of treatments received.

	Total		TNM7 Stage									
			I		II		III		IV		Unknown	
	n	(%)	n	(%)	n	(%)	n	(%)	n	(%)	n	(%)
**Total**	**353**	**(100.0)**	**68**	**(100.0)**	**75**	**(100.0)**	**111**	**(100.0)**	**81**	**(100.0)**	**18**	**(100.0)**
Untreated	28	(7.9)	2	(2.9)	1	(1.3)	1	(.9)	18	(22.2)	6	(33.3)
Only surgery	86	(24.4)	38	(55.9)	30	(40.0)	8	(7.2)	6	(7.4)	4	(22.2)
Only chemotherapy	14	(4.0)	0	(.0)	0	(.0)	0	(.0)	14	(17.3)	0	(.0)
Only radiotherapy	11	(3.1)	2	(2.9)	0	(.0)	4	(3.6)	3	(3.7)	2	(11.1)
Surgery+Chemo	26	(7.4)	1	(1.5)	3	(4.0)	13	(11.7)	9	(11.1)	0	(.0)
Surgery+Radio	16	(4.5)	0	(.0)	8	(10.7)	7	(6.3)	1	(1.2)	0	(.0)
Chemo+Radio	10	(2.8)	2	(2.9)	3	(4.0)	4	(3.6)	1	(1.2)	0	(.0)
Surgery+Chemo+Radio	135	(38.2)	22	(32.4)	29	(38.7)	71	(64.0)	11	(13.6)	2	(11.1)
Target treatment*	17	(4.8)	0	(.0)	0	(.0)	0	(.0)	17	(21.0)	0	(.0)
Unknown	10	(2.8)	1	(1.5)	1	(1.3)	3	(2.7)	1	(1.2)	4	(22.2)

(*) 100% combined with chemotherapy; 82% combined with surgery; 41% combined with radiotherapy

Table S4. Observed and net survival of colorectal cancer patients at 1,3 and 5 years since diagnosis.

		Years since diagnosis									RER	95% CI	p-value
		1 year			3 years			5 years					
		OS	NS	95% CI	OS	NS	95% CI	OS	NS	95% CI			
**Total**		**76.7**	**79.1**	**(76.3 - 81.6)**	**59.9**	**65.9**	**(62.5 - 69.1)**	**51.9**	**62.2**	**(58.4 - 65.8)**	**-**		**-**
Gender	Male	75.6	78.3	(74.6 - 81.5)	56.3	62.8	(58.2 - 66.9)	48.5	58.8	(53.7 - 63.4)	1		-
	Female	78.5	80.4	(75.9 - 84.1)	65.5	70.8	(65.4 - 75.5)	57.2	67.6	(61.4 - 73.0)	0.73	(0.57 – 0.94)	0.015
Age group	<65	91.1	91.6	(88.1 - 94.2)	74.2	75.5	(70.4 - 79.8)	69.1	71.2	(65.9 - 75.9)	1		-
	65-74	78.3	79.7	(74.1 - 84.1)	66.5	70.3	(63.9 - 75.8)	55.9	61.8	(54.9 - 68.0)	1.47	(1.09 – 1.98)	0.012
	75+	64.2	68.8	(63.6 - 73.4)	44.3	55.5	(49.3 - 61.2)	35.8	55.2	(47.9 - 62.0)	2.37	(1.81 – 3.10)	<0.001
TNM7 Stage	I	92.8	95.1	(89.3 - 97.8)	85.6	92.3	(83.9 - 96.5)	79.6	90.4	(79.4 - 95.7)	1		-
	II	83.6	86.7	(81.4 - 90.6)	74.7	84.8	(77.8 - 89.7)	66.5	85.6	(76.4 - 91.4)	3.48	(1.21 – 9.92)	0.020
	III	88.8	91.6	(86.8 - 94.7)	69.3	75.9	(69.1 - 81.5)	61.7	72.7	(64.9 - 79.1)	5.32	(1.92 – 14.68)	0.001
	IV	49.1	50.0	(43.7 - 56)	20.6	21.6	(16.7 - 26.9)	10.5	11.2	(7.6 - 15.6)	44.87	(16.84 – 119.59)	<0.001
	Unknown	60.8	65.2	(48.5 - 77.7)	41.2	45.8	(28.4 - 61.6)	37.3	43.6	(25.1 - 60.8)	13.12	(4.27 – 40.32)	<0.001
T	T1	90.8	92.5	(84.4 - 96.5)	85.3	90.3	(79.9 - 95.5)	78.9	86.2	(73.5 - 93.1)	1		-
	T2	94.9	97.9	(82.6 - 99.8)	79.6	87.4	(74.3 - 94.1)	75.5	89.4	(71.2 - 96.4)	1.90	(0.35 – 10.40)	0.460
	T3	80.7	83.1	(79.4 - 86.2)	65.3	72.1	(67.5 - 76.2)	56.6	68.4	(63.1 - 73.1)	7.82	(1.85 – 33.08)	0.005
	T4	63.2	65.4	(57.7 - 72.1)	41.6	46.8	(38.5 - 54.6)	31.9	41.2	(32.4 - 49.8)	21.42	(5.03 – 91.01)	<0.001
	Tx	42.9	45.5	(34.4 - 56)	11.0	13.3	(6.7 - 22.1)	5.5	8.1	(2.8 - 16.9)	68.50	(16.07 – 292.05)	<0.001
N	N0	84.2	87.0	(83.1 - 90)	74.3	82.7	(77.8 - 86.7)	66.5	81.8	(75.6 - 86.5)	1		-

	N1	85.2	87.5	(81.8 - 91.5)	64.6	70.3	(62.8 - 76.5)	57.2	65.9	(57.6 - 73)	1.97	(1.33 – 2.92)	0.001
	N2/N+	72.0	73.9	(67.1 - 79.5)	45.8	49.6	(42.1 - 56.7)	35.0	41.3	(33.8 - 48.7)	4.72	(3.35 – 6.66)	<0.001
	Nx	40.9	42.8	(33.7 - 51.7)	19.7	21.0	(13.8 - 29.3)	15.0	15.8	(9.4 - 23.7)	12.37	(8.68 – 17.62)	<0.001
Charlson comorbidity index	No comorbidity (0-1)	85.6	87.9	(84.3 - 90.6)	74.4	81.1	(76.5 - 84.9)	67.3	79.7	(74.2 - 84.1)	1		-
	Low comorbidity (2)	77.8	80.6	(73.0 - 86.3)	62.3	69.0	(59.7 - 76.6)	54.5	65.1	(54.5 - 73.9)	1.57	(1.03 – 2.38)	0.034
	High comorbidity (3+)	63.8	66.2	(60.8 - 71.0)	38.4	43.2	(37.5 - 48.7)	29.2	36.4	(30.5 - 42.2)	4.72	(3.61 – 6.17)	<0.001

Table S5. Observed and net survival of colon cancer patients at 1,3 and 5 years since diagnosis.

		Years since diagnosis									RER	95% CI	p-value
		1 year			3 years			5 years					
		OS	NS	95% CI	OS	NS	95% CI	OS	NS	95% CI			
**Total**		**75.3**	**77.7**	**(74.1 - 80.8)**	**58.5**	**64.6**	**(60.3 - 68.5)**	**51.5**	**61.8**	**(57 - 66.2)**	**-**		**-**
Gender	Male	74.1	76.9	(72.1 - 80.9)	54.4	61.2	(55.6 - 66.3)	47.3	58.4	(52.1 - 64.2)	1		-
	Female	77.2	79.0	(73.3 - 83.7)	65.1	69.9	(63.1 - 75.6)	58.1	67.1	(59.5 - 73.6)	0.65	(0.48 – 0.89)	0.007
Age group	<65	89.2	89.7	(84.8 - 93.1)	71.3	72.5	(66.0 - 78.1)	67.3	69.4	(62.5 - 75.3)	1		-
	65-74	79.1	80.5	(73.6 - 85.7)	67.0	70.8	(62.9 - 77.4)	56.6	62.7	(54.1 - 70.1)	1.31	(0.91 – 1.86)	0.148
	75+	62.3	66.8	(60.4 - 72.4)	43.5	54.6	(47.1 - 61.5)	36.3	55.5	(46.4 - 63.6)	2.20	(1.59 – 3.05)	<0.001
TNM7 Stage	I	93.7	95.9	(87.3 - 98.7)	87.4	93.2	(81 - 97.7)	82.0	91.7	(76.2 - 97.3)	1		-
	II	83.7	86.7	(80.3 - 91.2)	74.4	83.8	(75.5 - 89.5)	67.5	84.6	(73.9 - 91.2)	7.62	(0.79 – 73.27)	0.079
	III	86.7	89.9	(83 - 94.2)	66.9	74.4	(65.1 - 81.7)	60.8	73.9	(63.1 - 82.1)	12.54	(1.33 – 118.34)	0.027
	IV	47.5	48.4	(40.8 - 55.6)	19.7	20.7	(15 - 27.1)	9.8	10.7	(6.5 - 16)	109.44	(11.94 – 1002.96)	<0.001
	Unknown	58.8	62.3	(41.7 - 77.4)	38.2	44.4	(24.3 - 62.7)	35.3	44.1	(21.3 - 64.8)	36.62	(3.68 – 363.91)	0.002
T	T1	91.8	93.3	(82.6 - 97.5)	86.3	89.9	(76.7 - 95.8)	82.2	88.6	(73.6 - 95.4)	1		-
	T2	98.3	101.3	(101.3 - 101.3)	86.7	94.5	(67 - 99.2)	80.0	92.6	(58.6 - 98.9)	1.55	(0.14 – 162.82)	0.853
	T3	79.4	82.0	(77.2 - 85.8)	63.4	70.5	(64.6 - 75.6)	56.4	69.0	(62.2 - 74.8)	22.61	(0.45 – 1125.10)	0.118
	T4	61.1	63.3	(53.8 - 71.4)	41.3	46.7	(36.7 - 56.1)	31.7	40.2	(29.9 - 50.4)	61.15	(1.22 – 3049.47)	0.039
	Tx	42.0	44.3	(31.6 - 56.2)	10.1	13.1	(5.9 - 23.3)	4.3	7.7	(2.1 - 18.3)	198.37	(3.98 – 9898.47)	0.008

N	N0	84.2	86.9	(82.3 - 90.4)	74.5	82.4	(76.5 - 87)	67.3	81.1	(73.8 - 86.6)	1		-
	N1	82.7	85.3	(77.7 - 90.4)	62.0	68.2	(58.6 - 76)	54.0	63.7	(53 - 72.6)	2.45	(1.52 – 3.94)	<0.001
	N2/N+	65.8	67.8	(58 - 75.8)	36.8	40.7	(31 - 50.2)	29.1	36.4	(26.5 - 46.3)	6.57	(4.26 – 10.13)	<0.001
	Nx	37.0	38.2	(27.2 - 49.1)	14.8	15.6	(8.4 - 24.8)	11.1	11.8	(5.6 - 20.6)	16.50	(10.65 – 25.46)	<0.001
Charlson comorbidity index	No comorbidity (0-1)	85.4	87.6	(83 - 91)	73.5	79.9	(73.9 - 84.6)	68.0	79.8	(72.9 - 85.2)	1		-
	Low comorbidity (2)	75.2	78.0	(68.2 - 85.1)	61.9	69.5	(57.9 - 78.4)	52.2	63.7	(50.4 - 74.4)	1.72	(1.04 – 6.69)	0.036
	High comorbidity (3+)	62.5	64.9	(58.3 - 70.8)	37.9	42.8	(36 - 49.5)	30.1	37.9	(30.7 - 45)	4.77	(3.40 – 6.69)	<0.001

Table S6. Observed and net survival of rectal cancer patients at 1,3 and 5 years since diagnosis.

		Years since diagnosis: 1 year			3 years			5 years			RER	95% CI	p-value
		OS	NS	95% CI	OS	NS	95% CI	OS	NS	95% CI			
**Total**		**79.8**	**82.3**	**(77.4 - 86.2)**	**63.1**	**69.1**	**(63.0 - 74.4)**	**53.1**	**63.5**	**(56.6 - 69.6)**	**-**		**-**
Gender	Male	79.4	82.2	(75.7 - 87.1)	60.7	66.5	(58.5 - 73.3)	51.4	59.9	(51.2 - 67.6)	1		-
	Female	80.4	82.5	(74.4 - 88.2)	66.7	73.2	(63.3 - 80.8)	55.8	69.0	(57.2 - 78.2)	0.94	(0.61 – 1.43)	0.768
Age group	<65	95.1	95.6	(89.6 - 98.2)	80.5	81.9	(73.4 - 87.9)	73.2	75.5	(66.3 - 82.5)	1		-
	65-74	76.7	78.0	(67.5 - 85.5)	65.6	69.2	(57.5 - 78.3)	54.4	60.1	(47.7 - 70.5)	1.91	(1.13 – 3.24)	0.015
	75+	68.3	73.2	(63.9 - 80.5)	46.0	57.7	(46.5 - 67.4)	34.5	55.0	(41.6 - 66.5)	2.85	(1.77 – 4.61)	<0.001
TNM7 Stage	I	91.2	93.8	(81.6 - 98)	82.4	90.6	(73.7 - 96.8)	75.0	87.7	(65.7 - 96)	1		-
	II	84.0	87.3	(75.4 - 93.7)	77.3	89.3	(71.8 - 96.2)	65.3	89.9	(61.2 - 97.7)	1.81	(0.54 – 6.07)	0.336
	III	91.9	94.1	(85.9 - 97.6)	73.0	78.2	(67.4 - 85.8)	63.1	70.9	(58.8 - 80)	2.51	(0.85 – 7.36)	0.095
	IV	53.1	54.2	(42.3 - 64.6)	23.5	24.2	(15.2 - 34.5)	12.3	12.9	(6.5 - 21.6)	19.18	(6.97 – 52.79)	<0.001
	Unknown	64.7	71.2	(38.7 - 88.5)	47.1	48.8	(17.2 - 74.6)	41.2	42.8	(14.1 - 69.2)	4.03	0.79 – 20.46)	0.092
T	T1	88.2	90.5	(71 - 97.1)	82.4	90.4	(63.1 - 97.8)	70.6	79.5	(49.7 - 92.8)	1		-
	T2	89.5	92.6	(72.5 - 98.2)	68.4	76.2	(54.3 - 88.6)	68.4	84.3	(53.8 - 95.4)	1.69	(0.34 – 8.34)	0.520
	T3	83.3	85.5	(79.2 - 90)	69.0	75.2	(67.3 - 81.5)	57.1	67.4	(58.4 - 74.9)	2.79	(0.72 – 10.81)	0.138
	T4	68.4	70.6	(56 - 81.1)	43.9	48.7	(33.5 - 62.3)	33.3	44.8	(27.9 - 60.3)	7.46	(1.89 – 29.45)	0.004
	Tx	45.0	49.3	(24.9 - 69.8)	15.0	15.3	(3 - 36.6)	10.0	10.2	(1.4 - 29.5)	22.54	(5.48 – 92.81)	<0.001
N	N0	84.2	87.2	(79.1 - 92.3)	74.4	84.2	(73.5 - 90.9)	64.7	84.0	(69.5 - 92)	1		-

	N1	89.9	91.8	(81.4 - 96.5)	69.6	74.1	(61.2 - 83.3)	63.3	70.1	(55.7 - 80.5)	1.27	(0.63 – 2.56)	0.508
	N2/N+	80.2	82.0	(72.1 - 88.7)	57.3	61.0	(49.3 - 70.8)	42.7	47.7	(36.1 - 58.5)	2.91	(1.64 – 5.16)	<0.001
	Nx	47.7	51.0	(34.4 - 65.4)	29.5	32.0	(16.8 - 48.3)	22.7	24.0	(10.7 - 40.1)	6.96	(3.71 – 13.06)	<0.001
Charlson comorbidity index	No comorbidity (0-1)	86.7	89.2	(82.9 - 93.2)	76.6	83.8	(75.6 - 89.4)	66.5	80.0	(69.8 - 87.0)	1		-
	Low comorbidity (2)	82.7	85.4	(70.7 - 93.1)	63.5	68.7	(50.8 - 81.2)	59.6	68.4	(48.7 - 81.8)	1.35	(0.63 – 2.86)	0.441
	High comorbidity (3+)	67.0	69.3	(59.2 - 77.3)	40.2	44.6	(34.2 - 54.5)	27.7	33.5	(23.6 - 43.8)	4.69	(2.98 – 7.38)	<0.001

Table S7. Observed (OS) and net survival (NS) at 1,3 and 5 years since diagnosis of **stage II/III colorectal cancer** patients and relative excess risk of death (RER) as a function of adherence to the quality indicators (QIs).

		Years since diagnosis									RER		p-value
		1 year			3 years			5 years					
		OS	NS	95% CI	OS	NS	95% CI	OS	NS	95% CI			
QI1	No	75.0	78.1	(58.8 - 89.1)	61.1	69.0	(46.5 - 83.6)	55.6	70.4	(43.5 - 86.2)			
	Yes	87.1	90.0	(86.6 - 92.7)	72.9	81.3	(76.5 - 85.2)	64.8	79.9	(74 - 84.6)	0.73	(0.27 – 1.96)	0.536
QI2	No	77.0	80.3	(72.9 - 85.8)	63.4	73.3	(64.2 - 80.5)	56.3	76.6	(65.1 - 84.8)			
	Yes	90.7	93.5	(89.6 - 95.9)	76.3	83.8	(78.2 - 88.1)	68.0	80.4	(73.7 - 85.6)	0.54	(0.34 – 0.87)	0.011
QI3	No	89.2	92.6	(85.7 - 96.2)	78.9	88.7	(78.8 - 94.2)	71.7	89.9	(76.4 - 95.9)			
	Yes	85.9	89.0	(83.9 - 92.6)	70.4	79.2	(72.3 - 84.6)	63.5	80.5	(71.8 - 86.7)	2.30	(0.99 – 5.32)	0.053
QI4	No	94.0	96.1	(83.5 - 99.1)	77.6	83.3	(69 - 91.4)	67.2	75.3	(59 - 85.9)			
	Yes	92.3	93.9	(69.2 - 98.9)	73.1	77.8	(53 - 90.6)	61.5	66.0	(40 - 82.9)	1.28	(0.45 – 3.62)	0.646
QI5	No	97.3	99.8	(0 - 100)	82.2	89.4	(74.4 - 95.9)	74.0	86.8	(68.7 - 94.8)			
	Yes	97.6	99.5	(29.2 - 100)	81.9	86.9	(77.6 - 92.5)	75.6	83.8	(72.8 - 90.6)	0.87	(0.35 – 2.18)	0.763
QI6	No	86.8	90.0	(81.2 - 94.8)	70.2	79.4	(67.6 - 87.2)	64.0	82.8	(67.1 - 91.5)			
	Yes	89.4	92.4	(88.6 - 95)	75.9	84.4	(78.9 - 88.5)	67.7	82.9	(76.2 - 87.8)	0.89	(0.47 – 1.67)	0.713
QI7	No	68.0	74.3	(56.9 - 85.4)	42.0	55.4	(35.4 - 71.5)	38.0	62.0	(35.5 - 80.2)			
	Yes	95.6	97.5	(88.7 - 99.5)	77.9	83.0	(72.9 - 89.6)	70.8	79.3	(67.6 - 87.1)	0.33	(0.16 – 0.70)	0.004
QI8	No	79.0	82.7	(68.7 - 90.8)	69.4	81.2	(62.5 - 91.1)	58.1	84.0	(56.1 - 94.9)			
	Yes	93.5	95.7	(88.2 - 98.5)	77.4	83.4	(73.5 - 89.9)	66.9	75.8	(64.3 - 84.1)	0.92	(0.40 – 2.14)	0.848
Overall adherence (≥75% of indicators)	No	80.6	84.0	(77.8 - 88.6)	64.7	73.8	(65.8 - 80.2)	56.5	74.0	(64.3 - 81.4)			
	Yes	90.2	92.8	(88.6 - 95.5)	77.3	85.1	(79.1 - 89.4)	69.6	82.9	(75.6 - 88.1)	0.41	(0.25 – 0.67)	<0.001

Table S8. Peri-operative mortality as a function of key patient and treatment characteristics.

		Peri-operative mortality		
		n	(%)	P value
Gender	Male	33	(6.21)	0.658
	Female	18	(5.28)	
Age group	<65	4	(1.29)	<.001
	65-74	12	(4.96)	
	75+	35	(10.97)	
Charlson comorbidity index	No comorbidity (0-1)	14	(2.98)	<.001
	Low comorbidity (2)	11	(7.75)	
	High comorbidity (3+)	26	(10.00)	
Smoker	Yes. currently	6	(5.31)	0.534
	Yes. previously	16	(6.35)	
	No. never	18	(4.44)	
Treatment modality	Without treatment	45	(9.43)	<.001
	Adjuvant	0	(0.00)	
	Neo-adjunvant	6	(4.55)	
